# Swimming in Plastamination: Polylactic Acid (PLA) Nanoplastics Affect Bull Sperm Functions

**DOI:** 10.3390/ijms27167091

**Published:** 2026-08-07

**Authors:** Alessia Gloria, Massimo Venditti, Erwin Pavel Lamparelli, Maria Zelinda Romano, Giovanna Della Porta, Andrea Viggiano, Antonietta Santoro, Alberto Contri, Rosaria Meccariello

**Affiliations:** 1 Department of Veterinary Medicine, University of Teramo, Loc. Piano d’Accio, 64100 Teramo, Italy; agloria@unite.it (A.G.); acontri@unite.it (A.C.); 2 Department of Experimental Medicine, University of Campania “Luigi Vanvitelli”, 80138 Naples, Italy; massimo.venditti@unicampania.it; 3 Department of Medicine, Surgery and Dentistry, University of Salerno, 84081 Baronissi, Salerno, Italy; elamparelli@unisa.it (E.P.L.); gdellaporta@unisa.it (G.D.P.); aviggiano@unisa.it (A.V.); ansantoro@unisa.it (A.S.); 4 Department of Medical, Human Movement and Well-Being Sciences, University of Naples Parthenope, 80133 Naples, Italy; mariazelinda.romano@collaboratore.uniparthenope.it; 5 Interdepartmental Observatory for Scientific Health Initiatives for Environmental Legislation and Disease Prevention, SHIELD, University of Salerno, 84084 Fisciano, Salerno, Italy

**Keywords:** plastic contamination, micro and nano-plastics, polylactic acid (PLA), semen quality, bovine

## Abstract

Plastic contamination (plastamination) is one of the main challenges of the 21st century. Microplastics (MPs, 5 mm–1 µm) and nanoplastics (NPs, <1 µm) are mainly produced from the environmental degradation of plastic waste and enter the food chain, bypass biological barriers, and bioaccumulate in tissues where they exert toxic/inflammatory effects. In the male reproductive system, MP/NPs can cross the blood–testis barrier, impair spermatogenesis, and affect semen quality parameters. While biodegradable polymers like polylactic acid (PLA) may represent an ecofriendly alternative to carbon fossil polymers, their real effects in reproduction have been poorly investigated. In the present manuscript, the effects of increasing doses of rhodamine B-loaded PLA-NPs (170 ± 20 nm mean size) on the physiology of frozen–thawed bovine sperm were investigated using a computer-assisted sperm analyzer (CASA) and flow cytometry. Sperm kinetics revealed a significant effect of PLA-NPs on progressive motility at 60 min, with higher values at 200 and 300 µg/mL doses (*p* < 0.05 vs. control). Flow cytometry showed that PLA-NP exposure did not alter acrosomal integrity (PNA488+); however, the proportion of spermatozoa showing high mitochondrial potential (MitoDR+) was significantly reduced compared to controls at a 300 µg/mL PLA-NP dose after 120 min of incubation. The proportion of spermatozoa metabolically active (MitoDR+) with contextual membrane destabilization (M540+) was significantly lower in samples with 200 and 300 µg/mL PLA, independently of the incubation time. Finally, immunofluorescence analysis revealed the internalization of PLA-NPs within spermatozoa at the longest exposure time. In conclusion, PLA-NPs are internalized in spermatozoa, with specific localization in mitochondria, and slightly affect progressive motility mainly by mitochondrial interference and plasma membrane destabilization. Despite limited effects on post-thaw bull sperm motility even at high doses, plastamination warrants consideration, and further studies on the biological effects of biodegradable plastics are recommended to preserve sperm physiology.

## 1. Introduction

Currently, environmental plastic contamination (plastamination) is one of the biggest pollution-related issues. From 1950 to 2023, the annual production of plastics worldwide increased from approximately 100 to over 400 million metric tons due to the increasing demand for plastic to produce daily life goods [1]. In parallel, low or inefficient plastic recycling rates have caused plastic accumulation in the environment. Environmental plastamination comprises different types of plastics such as fossil-based conventional plastics [e.g., non-degradable polyethylene (PE), polystyrene (PS), and polyvinyl chloride (PVC) and biodegradable polycaprolactone (PCL) and polybutylene adipate terephthalate (PBAT)] and the more recently introduced bioplastics that are manufactured from renewable sources, also known as non-conventional plastics [e.g., non-degradable bio-polyethylene terephthalate (Bio-PET) and biodegradable polylactic acid (PLA) and polylactic co-glycolic acid (PLGA)] [2].

Environmental plastics are primarily classified by size, i.e., megaplastics (>50 cm), macroplastics (5–50 cm), mesoplastics (0.5–5 cm), microplastics (MPs, 5 mm–1 µm) and nanoplastics (NPs, less than 1 µm) [3,4]. Currently, close attention is being paid to MPs and NPs that can be intentionally produced (primary MPs) or that originate from the physical, chemical and biological degradation of larger plastic goods or waste in both marine and terrestrial environments (secondary MPs) [5]. Due to their small size, both MPs and NPs can enter the human body through ingestion, inhalation or skin contact. They can bypass biological barriers and enter cells, thereby inducing inflammation, endocrine dysregulation, oxidative stress, and cellular damage with toxic effects on mitochondria, lysosomes, and the endoplasmic reticulum. These effects ultimately lead to cell death and alterations in energy homeostasis, intestinal microflora, and nervous, immune, and reproductive tissues, among others [2,6]. Hence, the presence of MPs in several human-derived biological samples, such as stool [7], meconium [8], urine [9], semen [10,11], blood [12], breastmilk [8], sputum [13], and follicular fluid [14], as well as in tissues such as the placenta [15], gonads, brain, and lung [10,16,17,18,19,20] raises concerns about the human health risks associated with MP/NP exposure and tissue bioaccumulation.

Interestingly, increasing plastamination has coincided with a decline in human semen quality and a rise in global male infertility [21], which is idiopathic in most cases. Since several plasticizers, such as bisphenols or phthalates, strongly affect reproduction by acting as endocrine disruptors [22,23], the number of preclinical studies on the toxicity of MPs/NPs in mammals has significantly increased, with most studies over the last five years focusing on male reproduction. Most experiments have been carried out in mice or cell lines, and data concerning the reproductive toxicity of MNPs has been reported in several species (primarily rat and mouse, but also bovine, pig, lamb and human) [24,25,26]. However, particular attention must be paid to farm animals, since they are continuously exposed to MP/NPs through contaminated feed, water, and soil, and represent a critical node in the human food chain [27]. Livestock species such as cattle, swine, and poultry accumulate plastic particles in edible tissues [28] as well as in milk products and eggs [29,30]. This tissue-level bioaccumulation implies that human dietary exposure to MP/NPs may be substantially underestimated, since the consumption of animal-derived food products represents an additional and often overlooked source of contamination that adds to direct environmental exposure [27]. These findings highlight the need for safe alternatives, such as “eco-friendly” biodegradable plastics, and for the development of mitigation strategies. In this respect, PLA polymer, a bioplastic derived from renewable resources such as corn starch or sugarcane, plays a significant role in the global biodegradable plastic market. Nevertheless, the use of PLA poses concern for health due to limited studies on its potential adverse effects on cells and living organisms. Recent studies have reported the internalization of PLA-NPs in various cell types, including human Peripheral Blood Mononuclear Cells (PBMCs), HT-29 colon cancer cells, and C6 glioma cells [31]. Moreover, toxic effects on reproduction have been described in mice [32,33] and during zebrafish development [34]. In mice, PLA-NPs have also been associated with possible “Trojan horse” effects in combination with copper sulfate (CuSO_4_), mediated by oxidative stress and ferritinophagy in testicular tissue [35]. To the best of our knowledge, however, no studies have focused on the effect of PLA on ejaculated spermatozoa. In this respect, spermatozoa could be an interesting model due to their total dependence on the environmental conditions, allowing the study of the interaction between contaminants and cellular function.

In the present study, bovine spermatozoa were used as a well-established in vitro reproductive model to investigate the effects of increasing concentrations of rhodamine B-loaded PLA-NPs (mean size: 170 ± 20 nm) on frozen–thawed sperm physiology, assessing sperm kinetics, functional attributes and intracellular localization. Our aim was to provide the first characterization of the direct in vitro effects of this biodegradable polymer on bovine sperm at doses previously reported effective for cellular internalization, contributing to the evidence on the biological safety of ecofriendly plastics.

## 2. Results

### 2.1. Sperm Kinetic Attributes

**Figure 1:**
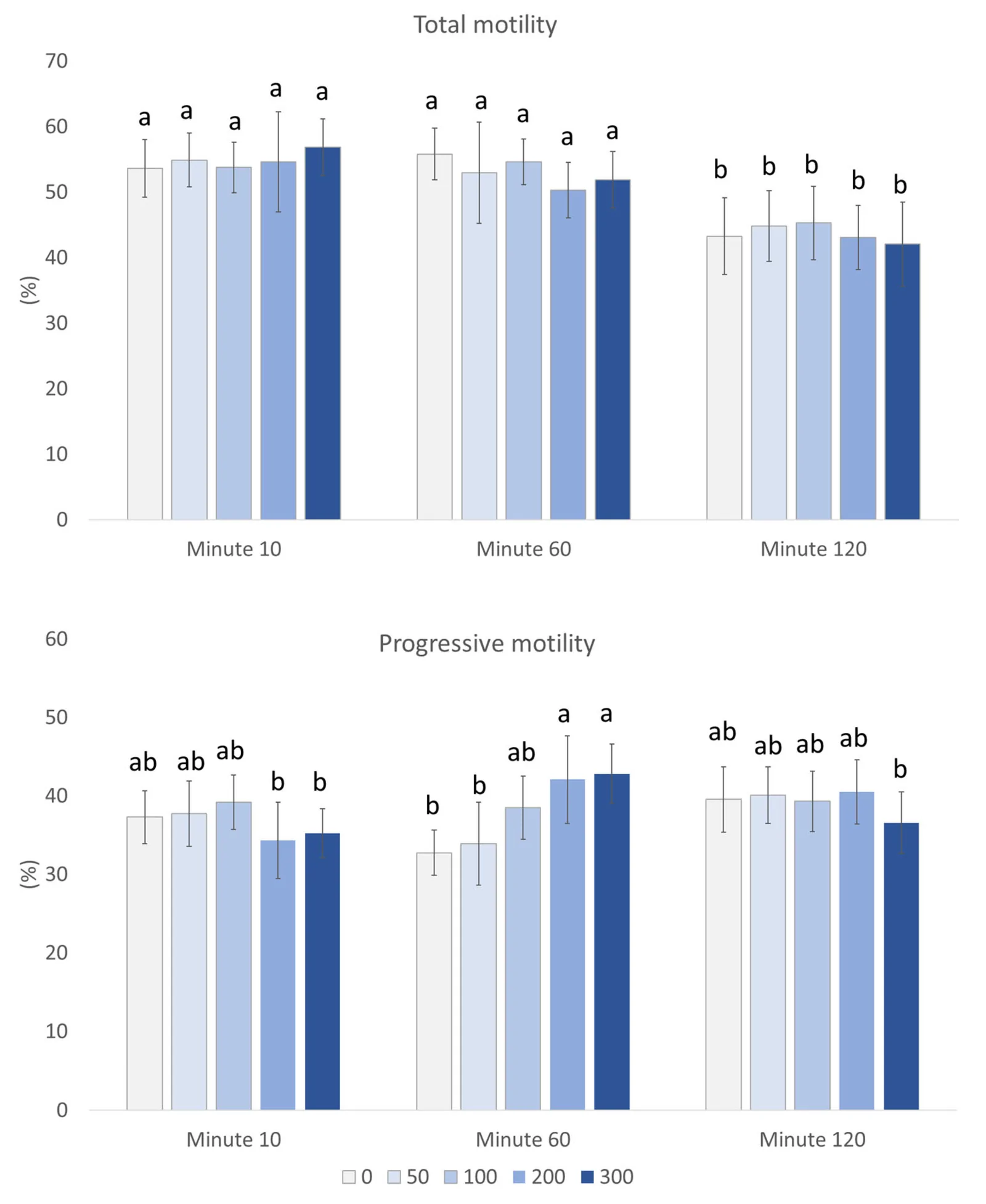
Graphical representation of the trend of total motility (%) and progressive motility (%) at different PLA-NP concentrations (0, 50, 100, 200, and 300 µg/mL) and times after thawing (10, 60, and 120 min). Different letters (a/b) indicate significant differences (*p* < 0.05).

Total motility (TM) in bovine cryopreserved spermatozoa showed similar values after thawing. As shown in Figure 1, no differences were found for TM among the different PLA-NP concentrations 10 min after thawing (*p* > 0.05). A significant decrease in TM was recorded, for all treatments, after 120 min of incubation (*p* < 0.05) compared with the values recorded after thawing. On the other hand, no differences were detected at 60 min of incubation (Figure 1). The concentration of PLA seemed to have negligible effects on the proportion of motile spermatozoa, with no differences among treatments (*p* > 0.05). Progressive motility followed the same pattern as total motility, with no significant differences among the tested PLA concentrations or treatment times. As shown in Figure 1, the values after 10 min after thawing were similar among the PLA-NP concentrations, with differences only found at 60 min; the sample without PLA showed lower values compared with PLA-NP concentrations of 200 and 300 (*p* < 0.05). When incubated with PLA-NP 50 and 100 µg/mL, the progressive motility showed intermediate values and was similar to all other treatments at 10 and 120 min of incubation (*p* > 0.05). Velocities were less dependent on PLA-NP concentration and treatment time since similar values were recorded at 10, 60, and 120 minutes for average path velocity (VAP), straight-line velocity (VSL), and curvilinear velocity (VCL). The flagellar beat frequency (BCF) appeared negligibly affected by the PLA, with a trend similar to the amplitude of lateral head displacement (ALH). The lack of an apparent effect of the PLA on kinetic sperm attributes at all PLA doses resulted in no significant differences in the progressiveness indexes (straightness (STR), linearity (LIN), and wobble (WOB)).

### 2.2. Multiparametric Sperm Function Assessment

**Figure 2:**
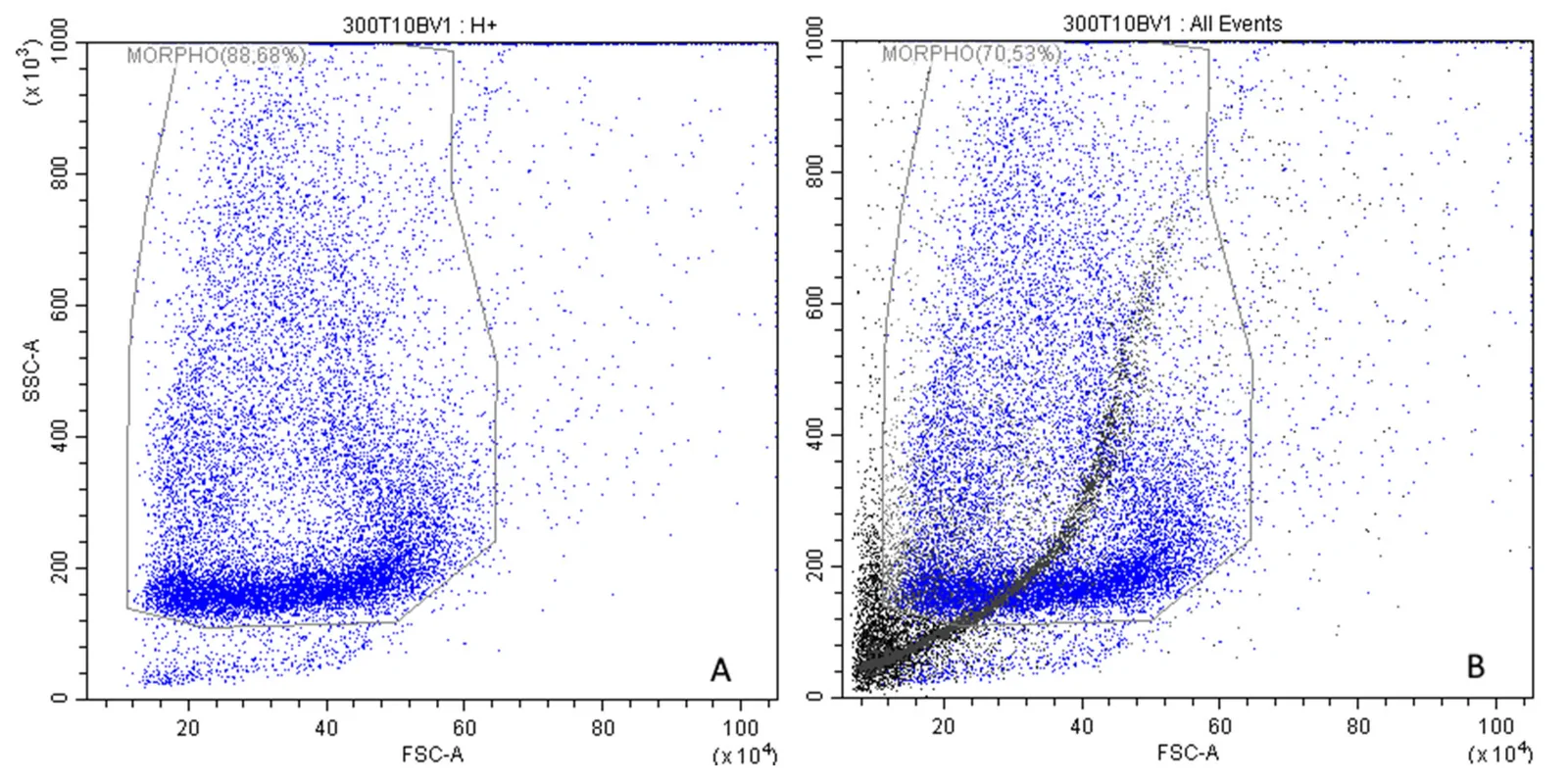
Representative cytograms of morphological gate-based events on forward scatter (FCS) and side scatter (SSC) with (**A**) or without (**B**) DNA gate, using Hoechst 33342 in a bull sample 10 min after thawing at a concentration of 300 µg/mL PLA-NPs. The blue-gated events represent events morphologically compatible with DNA-bearing spermatozoa.

In the present study, the gate created on events containing DNA appeared fundamental to discriminating spermatozoa. A DNA-based gating strategy, using Hoechst 33342 (H342), was implemented to selectively discriminate spermatozoa from non-cellular events, including PLA-NPs and debris (Figure 2). Notably, the total number of recorded events increased progressively together with the increase in PLA-NP concentration, reaching its maximum at 300 µg/mL, reflecting the growing contribution of fluorescent PLA-NPs to the overall signal. However, after applying the morphological and H342 gates, the number of DNA-positive events was comparable across all treatments (range: 34,692–36,919 events), confirming that the gating strategy effectively isolated spermatozoa and prevented particle-derived interference in the functional analysis.

A flow cytometry assessment of acrosomal integrity revealed no significant differences in the proportion of PNA488+ spermatozoa across PLA-NP concentrations or incubation times (*p* > 0.05), indicating that PLA-NP exposure does not impair acrosomal membrane integrity under the tested experimental conditions.

**Figure 3:**
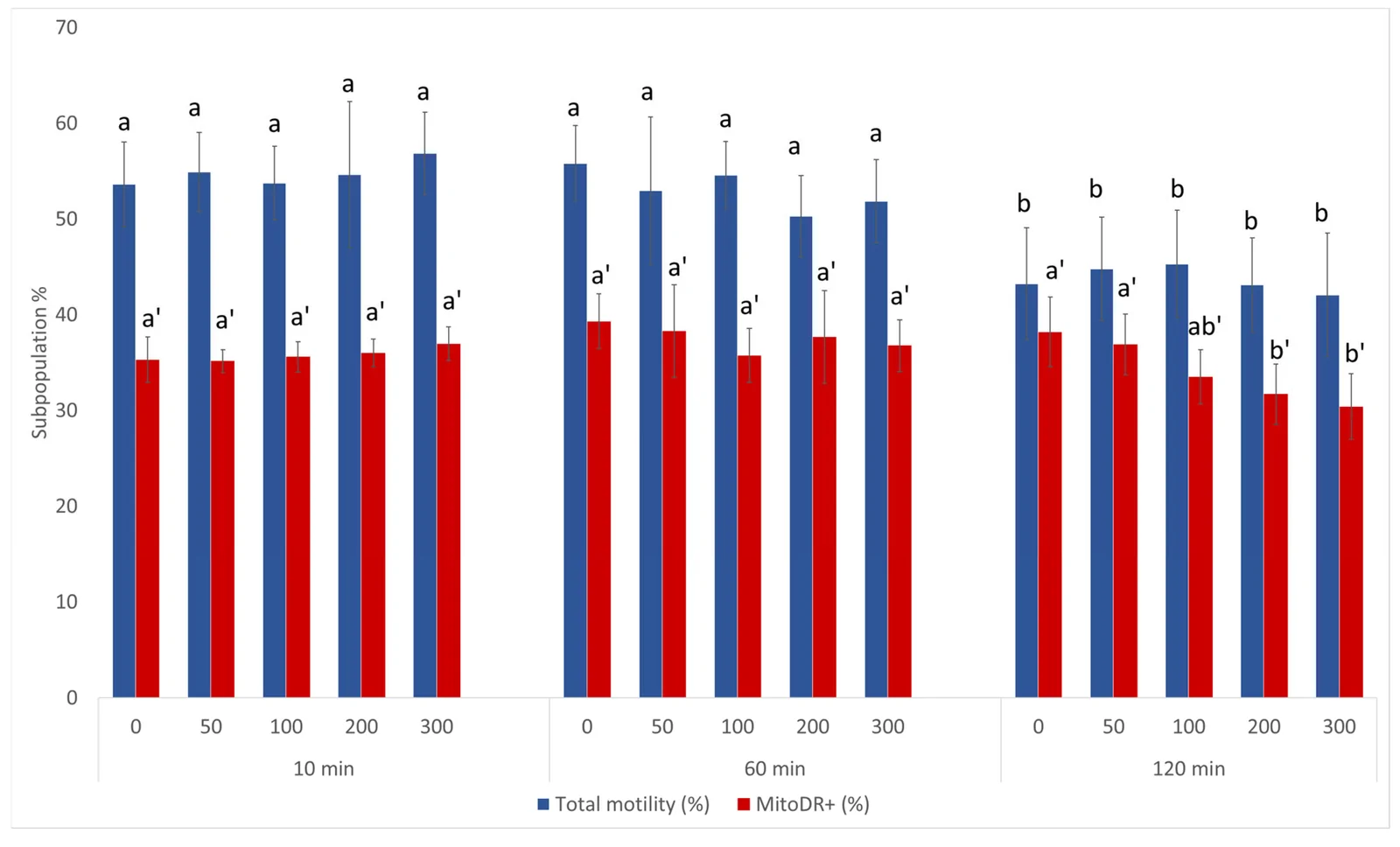
Graphical representation combining the trend of total motility and sperm subpopulation with mitochondrial activity (MitoDR+). Within the same attribute, different letters (a/b and a′/b′, respectively) refer to significant differences (*p* < 0.05).

Sperm subpopulations showing mitochondrial metabolism, assayed using Mitotracker Deep Red (MitoDR), were similar at the different PLA-NP concentrations at 10 and 60 min of incubation. At 120 min, the MitoDR+ subpopulation showed a PLA-related reduction that became significant at 300 µg/mL PLA-NPs (*p* < 0.05; Figure 3).

**Figure 4:**
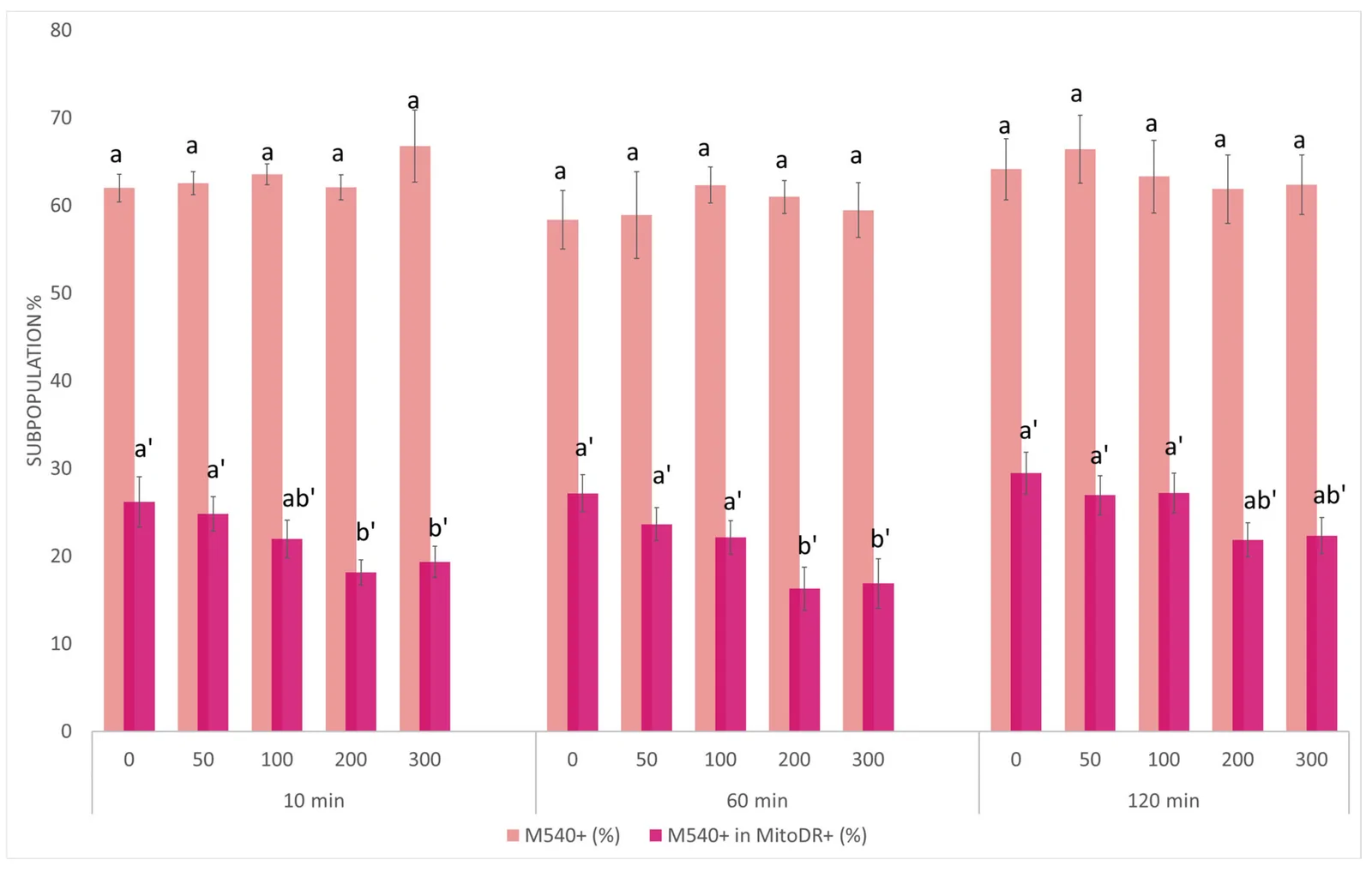
Graphical representation of the trend of the sperm subpopulation with membrane destabilization positive to merocyanine 540 (M540+), and the sperm subpopulation with membrane destabilization within the population of sperm with mitochondrial metabolism (M540+ in MitoDR+). Within the same subpopulation, different letters (a/b and a′/b′, respectively) refer to significant differences (*p* < 0.05).

The proportion of spermatozoa with plasma membrane destabilization, assayed using merocyanine 540 (M540), showed similar values at PLA-NP concentrations at all time points. A significant increase was detected at 120 min of incubation in the presence of 50 µg/mL PLA-NPs (*p* < 0.05). The samples incubated for 10 min at 300 µg/mL PLA-NPs showed a tendency of plasma membrane destabilization, but the level was not confirmed at 60 and 120 min of incubation, in which the values returned to those similar to the other treatment and the control (Figure 4). Interestingly, considering the proportion of spermatozoa with plasma membrane destabilization within the population of sperm with mitochondrial activity (M540+ in MitoDR+), the PLA seemed to exert a stabilization activity on the plasma membrane. Indeed, after thawing an assumed viable portion of spermatozoa with mitochondrial activity, plasma membrane destabilization was observed in all of the treatments. On the other hand, a progressive reduction in this subpopulation was detected at increasing PLA-NP concentrations, with significant differences at 200 and 300 µg/mL compared with controls, especially after 10 and 60 min of incubation (*p* < 0.05; Figure 4). At 120 min of incubation, the stabilization of the plasma membrane was less evident due to the possible confounding impact of the destabilizing role of cryopreservation on the membranes.

### 2.3. PLA-NP Internalization in Bull Spermatozoa

#### Intracellular Distribution

**Figure 5:**
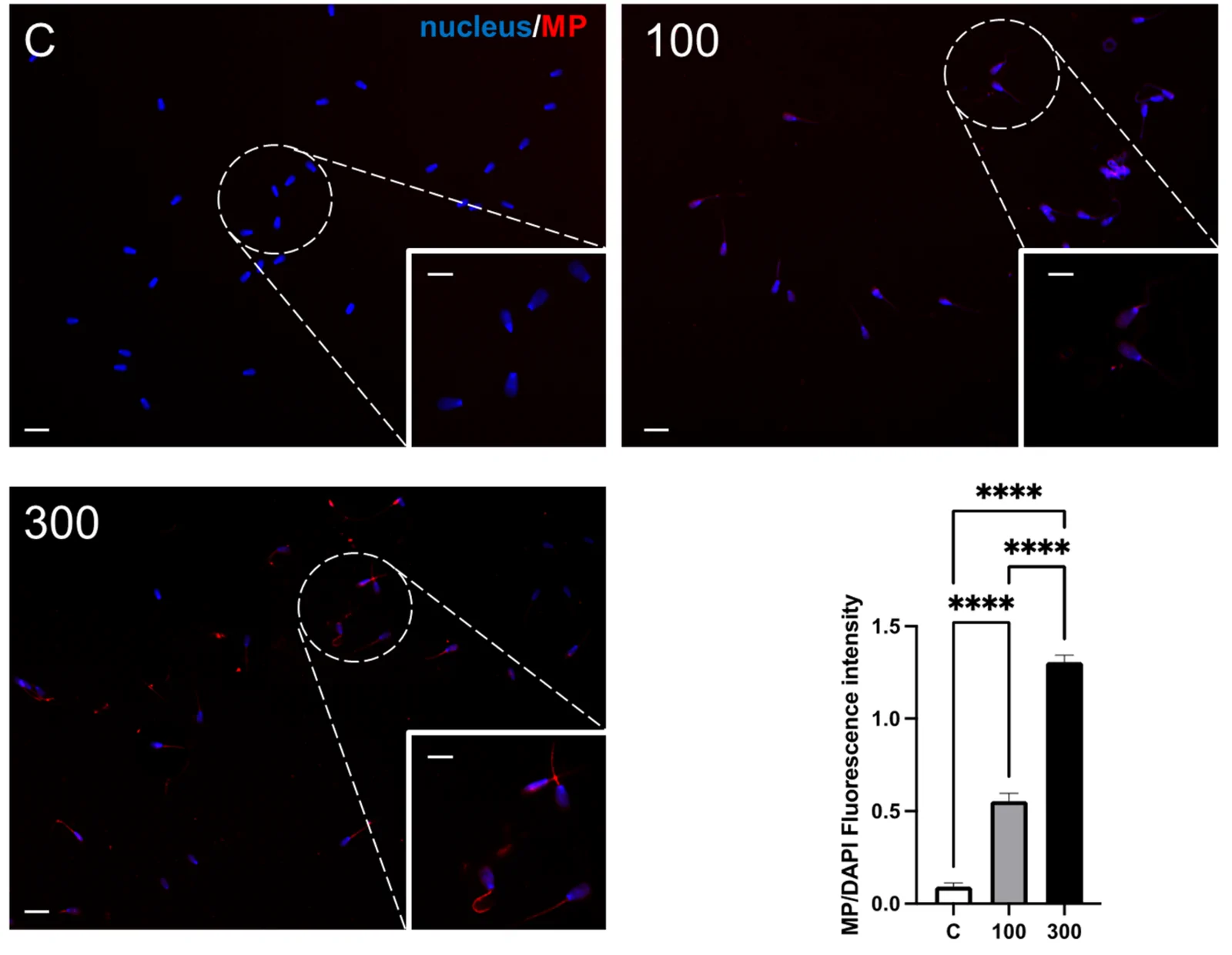
Subcellular localization of PLA particles in bull spermatozoa. Representative immunofluorescence images showing the localization of rhodamine-labeled PLA particles (red) in spermatozoa. The slides were counterstained with DAPI-fluorescent nuclear staining (blue). The images were captured at ×20 (scale bars = 20 µm) and ×40 for the insets (scale bars = 10 µm). The dashed circle indicates the area shown at higher magnification in the inset. The histogram shows the quantification of the fluorescence signal intensity. C, control; 100 and 300 indicate spermatozoa exposed to 100 and 300 µg/mL PLA particles, respectively. All values are expressed as means ± SEM from three animals in each group. ****: *p* < 0.0001 vs. control.

Although the intracellular amount of PLA-NPs was not directly quantified, fluorescence signal intensity was measured as an indirect semiquantitative indicator of PLA-NP accumulation within spermatozoa. Figure 5 shows that PLA-NPs were internalized by spermatozoa, with rhodamine fluorescence (red), primarily localized in the head region and, more prominently, in the midpiece where the mitochondria sheet is localized. Immunofluorescence analysis demonstrated a dose-dependent accumulation of PLA-NPs, with significantly higher signal intensity observed in the 300 µg/mL PLA-NP-treated group compared to both the control (*p* < 0.0001) and the 100 µg/mL PLA-NP (*p* < 0.0001) groups.

**Figure 6:**
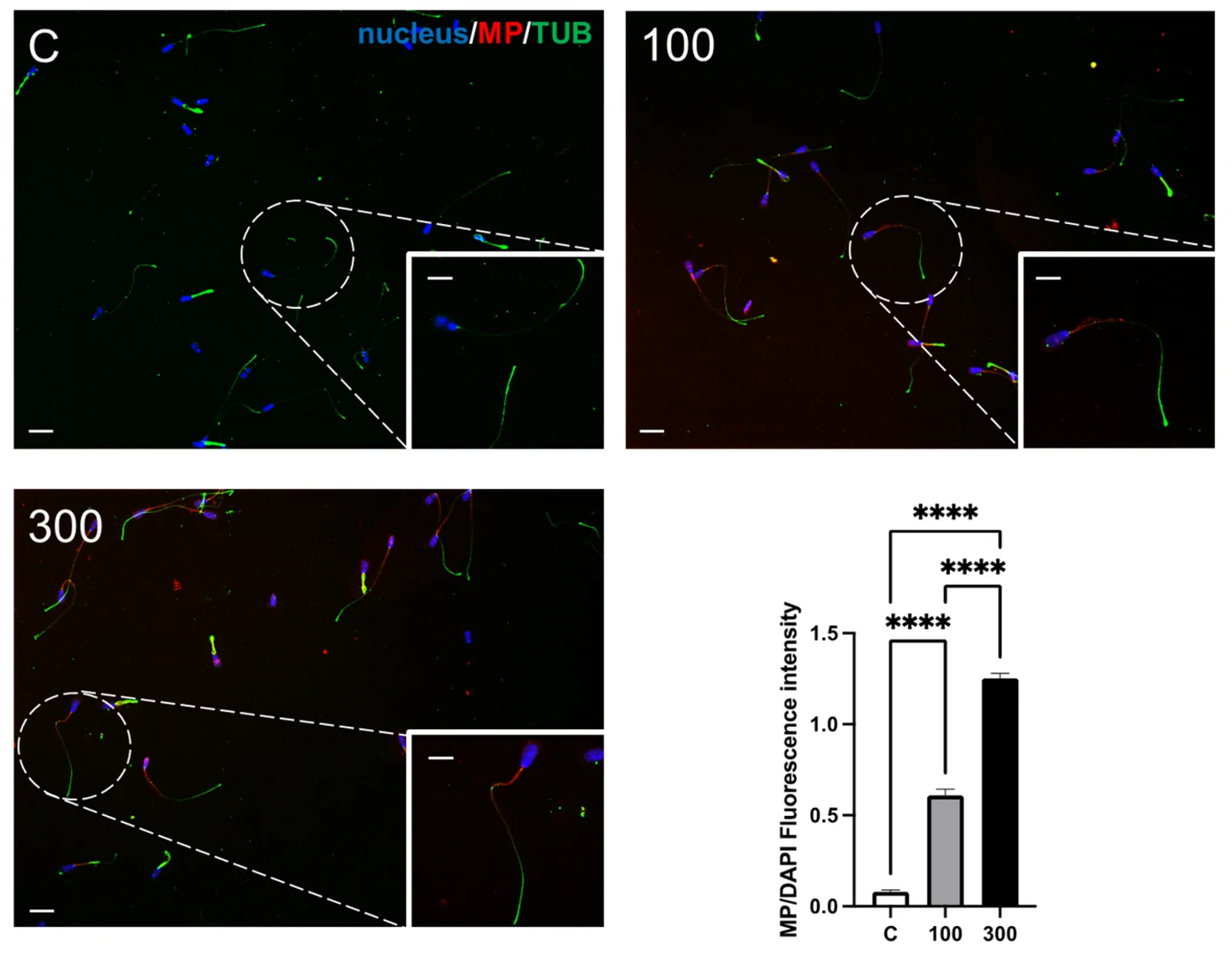
Subcellular localization of PLA particles and α-tubulin in bull spermatozoa. Representative immunofluorescence images showing the localization of rhodamine-labeled PLA particles (red, MP) and α-tubulin (green, TUB) in spermatozoa. The slides were counterstained with DAPI-fluorescent nuclear staining (blue, nucleus). The images were captured at ×20 (scale bars = 20 µm) and ×40 for the insets (scale bars = 10 µm). The dashed circle indicates the area shown at higher magnification in the inset. The histogram shows the quantification of the fluorescence signal intensity. C, control; 100 and 300 indicate spermatozoa exposed to 100 and 300 µg/mL PLA particles, respectively. All values are expressed as means ± SEM from three animals in each group. ****: *p* < 0.0001 vs. control.

These results were further corroborated by co-localization analysis using α-tubulin as a marker for the sperm flagellum. As shown in Figure 6, the spatial distribution pattern and fluorescence intensity of PLA-NPs closely matched those mentioned above, confirming their subcellular localization within the spermatozoa.

## 3. Discussion

Plastamination has now become one of the most pressing environmental issues and is further aggravated by the health risk arising from MPs and NPs. Long underestimated, these particles are now recognized as a growing global concern. Interestingly, the increasing accumulation of plastics in the environment [1] has coincided with the increase in global male infertility [21], which is idiopathic in most cases. Environmental factors including plastic additives have been shown to act as endocrine disruptors, thus exerting deleterious effects on spermatogenesis and semen parameters [22,23]. Studies in animal models have only recently focused on MPs/NPs, using commercially available plastic beads—mainly fluorescent PS-MPs—and demonstrated intratesticular bioaccumulation and deleterious effects on spermatogenesis and semen quality parameters [36,37,38,39]. The oxidative stress-mediated genotoxicity and cytotoxicity of PS-MPs, with possible implications for fertilization, have also been reported in human spermatozoa [40]. Nevertheless, these experimental conditions remain far from mimicking real environmental conditions in terms of plastic debris composition, shape, size or concentrations [2].

MP/NPs have also been reported in human testis and semen [10,11]; a cross-sectional study involving 113 male participants detected PE, PS, and PP in semen and urine samples and significantly associated polytetrafluoroethylene (PTFE) exposure with decreased semen quality [41].

Recently, studies reported the bioaccumulation of bioplastics in cell lines, developing zebrafish embryo, and mouse testis [31,32,33,34,35], moving attention toward the possible adverse effects of eco-friendly polymers developed to mitigate plastamination. Therefore, in the present study, we used bovine spermatozoa, a well-established model for reproductive studies, to investigate the in vitro effects of ecofriendly PLA-NPs on sperm kinematics and physiology, revealing mild but noteworthy effects on sperm physiology for the first time.

In bulls, Grechi et al. first detected twenty-four different micro-range plastic polymers in epididymal sperm at an average concentration of 0.37 µg/mL and with an average particle size of 17 ± 27.9 × 8.3 ± 9.8 µm [26]. Then, the authors tested a mixture of 1.1, 0.5, and 0.3 μm PS-MPs at environmentally relevant doses (0.02 and 0.9 μg/mL) in vitro for 2 h, revealing deleterious effects on sperm motility and fertilizing ability due to the binding of PS beads to the sperm surface [26]. PS-exposed sperm had significant impacts on in vitro fertilization, with increased oxidative stress and apoptosis in embryos and reduced blastocyst development [26]. Similarly, in a mouse model orally exposed to a mixture of MNPs of different size ranges, CASA analysis reported impaired sperm kinematics, with a decrease in progressive sperm motility, linearity and straight-line velocity of sperm movement, most pronounced in the smaller MNP size ranges (25–30 nm and 1–5 µm) [38]. In the present study, we incubated thawed bovine semen with increasing doses of PLA-NPs, revealing that this biopolymer is not toxic to spermatozoa. However, we report quite negligible effects of semen parameters at low, medium and long exposure times (respectively 10′, 60′ and 120′), but significant effects on progressive sperm motility at all tested doses after 60′ exposure. Although fluorophore-specific effects on membrane/mitochondrial probes cannot be formally excluded, the rhodamine-B-loaded PLA-NPs used in the present study have been previously qualitatively validated in terms of encapsulation efficiency, cytotoxicity, and cellular uptake, in parallel to the cytotoxicity of rhodamine-B alone used as a negative control [31].

Recently, Grechi et al. provided no evidence of PS beads being internalized by sperm [26], while our present data revealed that PLA particles in the nano range (~170 nm) had been localized by IFL in sperm heads and tails with dose-dependent trends.

Additionally, recent findings from in vivo studies reported the accumulation of PLA-MPs in Leydig cells along with inhibited autophagy and mitophagy and accelerated senescence [33], and in mouse spermatozoa as well [32]. Following oral exposure to PLA-MPs for 28 days, and after degradation in the digestive system, these PLA-MPs crossed the blood–testis barrier and induced mitochondrial dysfunction in the testicular microenvironment with mechanisms involving the production of reactive oxygen species (ROS) and oxidative stress. As a consequence, sex hormone levels decreased and the physiology and morphology of mature gametes were resultingly impaired, with spermatozoa exhibiting low motility, low concentration, and a higher deformity rate [32]. Focusing on spermatozoa, Zhao and coworkers [32] demonstrated PLA-MP internalization in the mitochondrial sheath of spermatozoa, with impairment in mitochondrial function and disruption of the mitochondrial structure [32]. The proposed in vitro model does not parallel chronic exposure in terms of long-term effects, bioaccumulation, or systemic effects, which could exacerbate the impact of NPs on overall sperm quality. The data presented in this study revelated a negligible effect of PLA-NPs on sperm’s structural membranes, as demonstrated by the constant level of acrosome reaction (PNA488+ spermatozoa) in the samples at all concentrations and incubation times. Nevertheless, in the present study, functional data linked PLA-NP exposure to mitochondrial activity. In fact, the MitoDR+ sperm subpopulations showing mitochondrial metabolism were reduced at the longer exposure time (120 min), with significant differences at the highest exposure dose (300 µg/mL PLA). Consistently, the immunofluorescence analysis of spermatozoa collected at the same exposure time revealed the presence of PLA-NPs in both head and tail, but prominently in the midpiece where the mitochondria sheet is localized. Curiously, after thawing, PLA-NPs at 200 and 300 µg/mL seem to exert protective effects on membrane destabilization, but only in metabolically active sperm, once again linking PLA-NP exposure to sperm metabolism. Further molecular and metabolomic analysis will be conducted in future work to corroborate present data and to mechanistically explain the effects of PLA on sperm. Nevertheless, current preliminary investigations on the direct effect of bioplastics on semen parameters and spermatozoa deserve attention and open new perspectives on the modulation of sperm quality by environmental pollutants.

From this preliminary study on the direct effects of PLA-NPs on spermatozoa, several unanswered questions arise, primarily related to the fate of PLA-NPs in spermatozoa and their possible interference in sperm metabolism.

In this respect, several hypotheses can be made. Glucose metabolism is essential for continued spermatogenesis and for the reproductive potential of sperm cells. While spermatozoa directly use glucose as a preferred energy source, lactate is the central energy substrate produced by Sertoli cells for germ cells [42]. Lactate exerts several critical functions in spermatogenesis and sperm physiology, including effects on motility (e.g., the shift from glucose-dependent hypermotility to lactate-dependent linear motility), on sperm intracellular pH and calcium with effects on capacitation and acrosome reaction [43]. In particular, in accordance with present data, in bull, lactate enhances the linear motility of spermatozoa by suppressing glucose incorporation in the midpiece and tail regions and shifting sperm metabolism toward oxidative phosphorylation of the respiratory chain (OXPHOS) [44]. Thus, present data might suggest that PLA mimics/interferes with the physiological activity of lactate. Disorders in lactate metabolism often result in cellular dysfunctions associated with male infertility [45,46]. Consistently, the testis-specific lactate dehydrogenase variant, LDH-C4, is a marker of sperm motility located in the middle and principal parts of mature spermatozoa, and it is a novel biomarker of infertility and cancer [47,48,49]. Nevertheless, recent evidence suggests that lactate also serves as a multifunctional signaling molecule with non-metabolic activities such as protein lactylation, the lastly discovered epigenetic modification occurring in germ cells at meiosis prophase I [50]. Recently, lactylated proteomic analysis revealed functional implications in asthenozoospermia [51], with 220 lactylated proteins related to the sperm glycolytic pathway and motility. A further hypothesis to be tested in the future is the possible impact of PLA-NPs in the remodeling of the epigenetic signature of germ cells and sperm proteome. Lastly, all of the open issues mentioned above have a common denominator: the still-unresolved question of the possible fate of PLA-NPs in sperm. Sperm mitochondria and cytoplasm contain LDH-C4, which reconverts lactate back to pyruvate within the mitochondrial matrix [52]. Hence, if PLA-NP degradation occurs, new biologically active metabolites (e.g., lactate and, consequently, pyruvate following the LDH-C4-catalyzed inter-conversion of pyruvate and lactate) may accumulate in sperm, thereby affecting their metabolic status and functions. The kinematics data revealed a PLA-NP-dependent increase in progressive motility, suggesting the possible degradation of PLA-NPs and changes in lactate levels. The increase in lactate could induce a mitochondrial boost, as lactate is a natural substrate of male germ cells [42]. On the other hand, the present data demonstrated that a longer exposure to PLA-NPs resulted in detrimental effects on mitochondrial activity, as demonstrated by the decrease in this sperm attribute at 120 min of incubation with the higher concentration (300 µg/mL). Increasing the PLA-NP exposure time could result in an over-production of ROS in the mitochondrial sheath, similar to other in vitro acute effects of nanoplastic toxicity [40].

A limitation of this study is related to the inability to completely evaluate the sample without plastic in in vitro models. Several disposable laboratory compounds are made from plastic; thus, it is unrealistic to consider PLA-NPs as the only plastic contaminant present in the samples. However, the starting conditions were the same for all treatments reported in this study; thus, the variations between PLA-NP concentrations and time of incubation indicate that the specific effects of the synthesized PLA-NPs can be successfully differentiated over any baseline laboratory contamination.

In conclusion, PLA-NPs do not exert toxic effects after ex vivo acute exposure on bovine spermatozoa, but enter sperm cells and slightly affect progressive motility and mitochondrial metabolism preserving plasma membrane destabilization in metabolically active cells. Noteworthily, this study was conducted deliberately on a single breed managed in uniform conditions to strictly control the confounding non-experimental factors. Despite the fact that PLA-NPs exert quite limited effects, even at high doses, on post-thaw bull sperm motility, these results could be considered the baseline from which trials on larger and heterogeneous subjects could be expanded to pave the way to a broad generalization of the results. The increasing diffusion of PLA-NPs in the environment could interfere with the physiological processes connected with reproduction, contributing to compromised reproductive outcomes in cattle breeding. Thus, bioplastics may be eco-friendly for the environment, but not fully exempt of risk for living organisms. Hence, for carbon fossil-derived plastic polymers, the biological effects of the so-called “green” polymers deserve further studies to assess the possible risk to reproductive health. The need for the development of sustainable mitigation strategies is also suggested.

## 4. Materials and Methods

### 4.1. Production of PLA NPs

PLA nanoparticles (PLA-NPs) were produced using a microfluidics-assisted nanoprecipitation method. Briefly, PLA (Resomer R 202, Evonik, Essen, Germany) polymer was dissolved in acetone at a concentration of 5 mg/mL for NPs. Milli-Q^®^ water supplemented with 1% polyvinyl alcohol (PVA) (molecular weight: 30,000–70,000, Aldrich Chemical Co., Milan, Italy) was used as the aqueous phase. Rhodamine B (No. 391, Radiant Dyes Laser, Wermelskirchen, Germany) was added to the organic phase at a concentration of 0.2 mg/mL to facilitate co-precipitation of the fluorescent tracer within the PLA-NPs. A microfluidic system (NanoGenerator Flex M, Precigenome LLC, San Jose, CA, USA) equipped with a passive staggered herringbone microchip was used to mix the organic and aqueous phases [31]. The tested organic-to-aqueous phase ratio was 1:1, with a total flow rate of 6 mL/min. The organic solvent in the NP suspension was removed by vacuum evaporation (Rotavapor R-100, BÜCHI, Flawil, Switzerland) at room temperature. The remaining surfactant was eliminated using ultracentrifugation, and the PLA-NPs were collected via filtration. For rhodamine B, the unencapsulated tracer in the supernatant was quantified using UV/Vis absorption spectroscopy (Multiskan GO spectrophotometer, Thermo Fisher Scientific, Waltham, MA, USA) at a peak wavelength of 550 nm. A maximum rhodamine B concentration of 0.2 mg/mL in the organic phase was selected to ensure sufficient fluorescence intensity for detection via confocal microscopy and flow cytometry analysis. Under these conditions, a maximum encapsulation efficiency of 55% was achieved, as determined by UV/Vis absorption spectroscopy of supernatants following NP suspension centrifugation. The mean particle size of PLA-NPs was 170 ± 64 nm.

### 4.2. PLA Preparation Media

PLA-NPs from a unique batch (exhibiting a maximum encapsulation efficiency of 55%), aliquoted and stored frozen to ensure consistency across all biological replicates, were suspended in TALP displaying a low capacitating effect (lcTALP) [53]. A working suspension of 500 µg/mL PLA-NPs was prepared. Dispersion was obtained by sonication using an ultrasound water bath at 37 kHz (100 W) for 10 min, in iced water. The working solution was used within 30 min of preparation.

### 4.3. Animals, Semen Collection and Preliminary Analysis

The effect of PLA on bovine sperm attributes was assessed in 13 cryopreserved semen batches, each obtained from a different adult Swiss Brown bull (n. 13). An a priori statistical power analysis, performed using G*Power software (version 3.1.9.7; Heinrich Heine University Düsseldorf, Düsseldorf, Germany), indicated that a minimum of 11 independent biological replicates was required to achieve a statistical power >80%. To compensate for potential sample loss or exclusion, a 20% oversampling margin was integrated into the study design, resulting in the final cohort of 13 bulls. The animals, aged between 3 and 6 years, were owned by the Superbrown Consortium (Bozen, Italy). The bulls were farmed in the Alpenseme AI Center of Trento (Ton, TN, Italy) and managed by the Provincial Breeders Federation of Trento (Italy). Notably, this study was intentionally conducted on a single breed managed under uniform environmental conditions and a highly standardized semen preparation protocol. While this homogeneous cohort might limit the immediate generalizability of the findings across diverse breeds or management systems, it was deliberately chosen to minimize confounding non-experimental factors that could otherwise modulate sperm attributes. Animals were handled for the commercial production of bovine insemination doses, according to good practice guidelines and the Italian Law for Welfare in Animal Production (D. Lgs. N. 146, 26 March 2001). After the insemination dose production, part of the batch was donated to perform the present study. For this reason, no ethical issues arose or needed to be disclosed when performing the present research.

Semen was collected from the bulls using an artificial vagina and was preliminarily examined for volume, concentration, subjective sperm motility, and morphology. The volume of the semen was estimated by weight using a CP6201 precision balance (Sartorius AG, Gottingen, Germany), assuming 1 mL = 1 g [54]. Sperm concentration was assessed using a photometer (Accucell, IMV Technologies, L’Aigle, France) after diluting the samples using a ratio of 1:100 with a sterile saline solution. Subjective motility, as mass motility, was estimated displacing 10 µL of diluted sample (1:30 v:v in prewarmed BioXcell—IMV Technologies) on a slide, covered with a coverslip and examined through a phase contrast microscope (Olympus BX 51, Olympus Europa SE & Co. KG, Hamburg, Germany) equipped with a heat plate set at 38 °C (magnification of ×400). Sperm morphology was assessed after dilution to 50 × 10^6^ sperm/mL with a 0.9% NaCl solution containing 3% glutaraldehyde [55]. The sample was then examined using a phase contrast microscope at ×1000 magnification. Only ejaculates with concentration >400 × 10^6^ sperm/mL, mass motility >75%, and normal sperm morphology >80% were cryopreserved.

For deep-freeze cryopreservation, semen was diluted to 100 × 10^6^ sperm/mL with Bioxcell (IMV Technologies), equilibrated for 3 h at 5 °C in a passive refrigerator, and packaged in 0.25 mL straws. The straws were then frozen using the Digicool 5300 programmable liquid nitrogen freezer (IMV Technologies), with the standard cooling rate (−5 °C/min from +4 °C to −10 °C; −40 °C/min from −10 °C to −100 °C; and −20 °C/min from −100 °C to −140 °C) reported in the literature [56]. Finally, the straws were plugged into liquid nitrogen for storage.

### 4.4. Experimental Design for the Effect of PLA on Bovine Spermatozoa

Each sample (8 straws/sample) was thawed in a water bath at 38 °C for 60 s, transferred in a 6 mL sterile plastic tube and further equilibrated for 10 min at the same temperature.

The thawed semen was washed 3 times with lcTALP, after subdivision into 5 aliquots. Then, the subaliquots were resuspended at 100 × 10^6^ sperm/mL into five aliquots and diluted with lcTALP with PLA-NPs at concentrations of 300 (PLA300), 200 (PLA200), 100 (PLA100), and 50 (PLA50) µg/mL. An lcTALP without PLA-NPs was used as a negative control (PLA0). After resuspension, the subsamples at different PLA-NP concentrations were incubated for 120 min. Sperm attributes (kinetic and sperm function parameters) were assessed at 10, 60, and 120 min of incubation at 38 °C.

### 4.5. Semen Assessment

#### 4.5.1. Objective Kinetics

Sperm kinetics were quantitatively evaluated using the Ceros II computer-assisted sperm analyzer (CASA, Hamilton Thorne Biosciences, Beverly, MA, USA). Each sample was warmed to 38 °C for 5 min, the concentration was adjusted to 30 × 10^6^ sperm/mL, and a 6 µL drop was then placed into a pre-warmed Makler chamber (Sefi Medical Instruments, Haifa, Israel) at this temperature. To ensure maximum objectivity and minimize potential observer bias, at least 10 fields per sample were selected at random and analyzed using fully automated software thresholds. A minimum of 1000 cells were analyzed to obtain the following parameters: total motility (TM, %), progressive motility (PM, %), average path velocity (VAP, μm/s), straight-line velocity (VSL, μm/s), curvilinear velocity (VCL, μm/s), amplitude of lateral head displacement (ALH, µm), beat cross frequency (BCF, Hz), straightness (STR, calculated as VSL/VAP, %), and linearity (LIN, calculated as VSL/VCL, %). The frame rate of the acquisition was 60 fps, and kinetic attributes were calculated on 45 frames. Spermatozoa with a VAP of 50 μm/s or greater and an STR of 80% or more were identified as progressive.

#### 4.5.2. Flow Cytometric Analysis

Flow cytometry (FC) multiparametric assessments were conducted utilizing the CytoFLEX flow cytometer (Beckman Coulter, San Jose, CA, USA), which is equipped with lasers operating at wavelengths of 405 nm (80 mW), 488 nm (50 mW), and 638 nm (50 mW). To ensure that only spermatozoa were included in the analysis, a morphological gate was established on the side scatter (SSC) versus forward scatter (FSC) scatter plot. Spectral compensation was executed by analyzing both non-stained and single-stained sperm samples. The sample was analyzed at 30 µL/min, and the event attributes were recorded after 15 s of analysis and stopped after 1 min of recording.

#### 4.5.3. Sperm Function by Multiparametric Flow Cytometry

For the multiparametric analysis, the concentration of different treatments was adjusted to 1 × 10^6^ sperm/mL. Each sample was stained with Hoechst 33342 (H342; Thermo Fisher Scientific, Waltham, MA, USA), peanut agglutinin conjugated to Alexa^®^488 (PNA-A488; Thermo Fisher Scientific, Waltham, MA, USA), merocyanine 540 (M540; Santa Cruz Biotechnology Inc., Dallas, TX, USA), and Mitotracker^®^ Deep Red (MitoDR; Thermo Fisher Scientific, Waltham, MA, USA). Events containing DNA were detected by H342 (excitation 405 nm; emission detected with a 450/45 band-pass filter) to discriminate between spermatozoa (with DNA) and other particles (mainly PLA NPs loaded with rhodamine B, but also other debris), as previously noted [57,58]. Acrosome integrity was evaluated using PNA conjugated to PNA-A488 (excitation 488 nm; emission detected with a 525/40 band-pass filter), assuming that positive events referred to damaged acrosome membrane; membrane organization was assessed using M540 (excitation 488 nm; emission detected with a 610/20 band-pass filter), assuming that positive events have membrane destabilization; mitochondrial activity was tested with MitoDR (excitation 638 nm; emission detected with a 660/10 band-pass filter), assuming that positive events have mitochondrial metabolism. A further sperm subpopulation was recorded as spermatozoa with membrane destabilization within the sperm subpopulation with cellular metabolism (M540+ in MitoDR+).

Each aliquot was incubated with H342 (10 nM final concentration), PNA-A647 (15 µg/mL final concentration), and MitoDR (1 nM final concentration) for 28 min at 38 °C. Merocyanine (2.7 µM final concentration) was added, and the sample was further incubated for an additional 2 min. The sample was analyzed using flow cytometry as described above.

### 4.6. Intracellular Distribution of PLA NPs

The intracellular distribution of PLA-NPs was evaluated subjectively by epifluorescence microscopy. A subsample of each PLA concentration (PLA300, PLA200, PLA100, PLA50, and PLA0), after 120 min of incubation, was spread on a slide, dried and post-fixed with 4% paraformaldehyde in PBS for 10′ at RT and then incubated with 0.1% Triton X-100 (#T8787; Sigma-Aldrich, Milan, Italy) in PBS. The slides were blocked with a PBS solution containing 20% normal goat serum (#NS02L Sigma-Aldrich, Milan, Italy) and 5% BSA (#05470; Sigma-Aldrich, Milan, Italy) before the addition of the anti-α-tubulin antibody (#E-AB-20036; Elabscience Biotechnology, Wuhan, China) diluted to 1:100 and incubated overnight at 4 °C. After washing with PBS, the slides were incubated for 1 h with Fluorescein (FITC) donkey anti-mouse IgG (#715-095-150; Jackson ImmunoResearch Europe Ltd., Ely, UK) diluted to 1:250 in the blocking solution. Slides were mounted with Vectashield +DAPI (#H-1200–10; Vector Laboratories, Peterborough, UK) for 5 min for nuclear staining and observed under a fluorescent microscope (Leica DM 5000 B + CTR 5000) with a UV lamp and saved using IM 1000 software (version 4.7.0; Leica Microsystems, Wetzlar, Germany). Photographs were taken on randomly selected fields using a Leica DFC320 R2 digital camera. To prevent any operator bias, a total of about 300 SPZ/slide was counted on these randomly selected fields, and the densitometric analysis of immunofluorescent signal intensity was performed with the Fiji plugin (version 3.9.0/1.53 t) of ImageJ software. All images were acquired using identical microscope and camera settings, and fluorescence quantification was performed using predefined and identical analysis settings for all samples.

### 4.7. Statistical Analysis

The different attributes were assessed for normal distribution using the Shapiro–Wilk test, assuming *p* < 0.05 as the threshold for normal distribution. Due to the normal distribution of all the variables, all values were presented as mean ± standard error of the mean (SEM).

The effect of the PLA-NPs on sperm attributes was assessed using a linear mixed model approach. The PLA-NP concentrations (300, 200, 100, 50, and 0 as control), the time of incubation (10, 60, and 120 min), and their interaction were defined as fixed factors, while the individual bull was included as a random effect (intercept) to account for the repeated-measures design and control for individual biological variation. The data expressed as percentages were converted into proportions (ranging from 0 to 1) and subjected to an arcsine square root transformation to stabilize variance (homoscedasticity) and normalize the residual distribution, aiming to satisfy the assumptions of linear parametric modeling that are inherently violated by bounded percentage data (0–100%). For the post hoc analysis, Bonferroni’s correction was adopted. The significance level was set at α = 0.05 (*p* < 0.05).

All statistical analyses and mixed-effects modeling were performed using Jamovi statistical software (version 2.3.21; The jamovi project, 2024; retrieved from https://www.jamovi.org), utilizing the GAMLj module for linear mixed models.

## Data Availability

The original contributions presented in this study are included in the article. Further inquiries can be directed to the corresponding author.
